# Effect of CeH_2.73_-CeO_2_ Composites on the Desorption Properties of Mg_2_NiH_4_

**DOI:** 10.3389/fchem.2020.00293

**Published:** 2020-04-15

**Authors:** Kaiyao Wu, Daqian Cai, Kaimei Shao, Tuguang Xue, Peng Zhang, Wei Li, Huai-Jun Lin

**Affiliations:** Institute of Advanced Wear and Corrosion Resistant and Functional Materials, Jinan University, Guangzhou, China

**Keywords:** Hydrogen storage materials, dehydrogenation, Mg_2_NiH_4_, CeH_2.73_/CeO_2_, catalysts

## Abstract

A series of CeH_2._73/CeO_2_ composites with different ratios of hydride and oxide phases are prepared from the pure cerium hydride via oxidation treatments in the air at room temperature, and they are subsequently doped into Mg_2_NiH_4_ by ball milling. The desorption properties of the as-prepared Mg_2_NiH_4_+CeH_2.73_/CeO_2_ composites are studied by thermogravimetry and differential scanning calorimetery. Microstructures are studied by scanning electron microscopy and transmission electron microscopy, and the phase transitions during dehydrogenation are analyzed through *in situ* X-ray diffraction. Results show that the initial dehydrogenation temperature and activation energy of Mg_2_NiH_4_ are maximally reduced by doping the CeH_2.73_/CeO_2_ composite with the same molar ratio of cerium hydride and oxide. In this case, the CeH_2.73_/CeO_2_ composite has the largest density of interface among them, and the hydrogen release effect at the interface between cerium hydride and oxide plays an efficient catalytic role in enhancing the hydrogen desorption properties of Mg_2_NiH_4_.

## Introduction

With the advantages of abundant natural resources and no pollution to the environment, hydrogen has been widely considered as an ideal carbon-free energy carrier. Hydrogen energy storage technology is a prerequisite for the large-scale utilization of hydrogen energy. Light-weight solid-state hydrogen storage materials have been considered to be ideal candidates for hydrogen storage because of the high hydrogen storage density and security consideration (Mohtadi and Orimo, 2016; Wang et al., 2016). Among the existing solid-state hydrogen storage materials, Mg-based hydrogen storage materials are widely studied and considered as promising solid-state hydrogen storage materials due to the high hydrogen storage capacity, abundance on the earth and low production cost (Ouyang et al., 2013, 2017; Rusman and Dahari, 2016; Shao et al., 2018).

MgH_2_ and Mg_2_NiH_4_ are two typical Mg-based hydrogen storage materials with hydrogen densities of 7.6 wt and 3.6 wt%, respectively (Reilly and Wiswall, 1968; Bogdanović, 1984; Liu et al., 2016, 2019; Zhan et al., 2016; Chen et al., 2018; Ding et al., 2019). Nevertheless, the stable thermodynamics and slow kinetics of hydrogen storage properties of Mg-based materials lead to harsh conditions for dehydrogenation (Bogdanović et al., 2010; Jain et al., 2010). The hydrogen desorption temperatures of MgH_2_ and Mg_2_NiH_4_ are usually as high as 250–350C. Many efforts have been made by researchers to improve the kinetics and reduce the desorption temperatures of MgH_2_ and Mg_2_NiH_4_. The commonly conducted methods include mechanical alloying, doping catalysts, nanostructuring, surface modification and so on (Terashita et al., 1999; Lin et al., 2012a, 2016; Shao et al., 2012; Eleskandarany et al., 2016; Zhang et al., 2017; Khan et al., 2018; Xu et al., 2019).

Doping catalysts by ball milling is a commonly-used and efficient method to enhance the hydrogenation/dehydrogenation kinetics of Mg-based materials (Ouyang et al., 2014a,b; Wang and Wang, 2017). To date, many additives have been explored, including oxides (Barkhordarian et al., 2003), transition metals (Hanada et al., 2005), hydrides (Ma et al., 2017), carbon-based materials (Lototskyy et al., 2013) and so on. It has been demonstrated that compounds with higher valences show higher catalytic effect on the hydrogen storage performances of Mg-based materials than that of the lower valence compounds (Bobet et al., 2002). Because of the unique 4*f* electron of the Ce element, Ce-based compounds are widely used in the catalysis field (Trovarelli, 1996). Long et al. (2013) reported Mg-Ce oxide powders produced by an arc plasma evaporation method. As a result, enthalpies of hydrogenation and dehydrogenation for Mg/MgH_2_ reduce to −71.0 and 75.4 kJ/mol H_2_, respectively. Moreover, the hydrogenation activation energy is reduced to only 47.75 kJ/mol. The composite can absorb 4.07 wt%-H at 323 K in 10 h. Their study indicates that minor addition of Ce oxide can remarkably improve the hydrogenation kinetics of Mg/MgH_2_. We previously reported that CeF_4_ was an efficient catalyst to enhance the hydrogen storage properties of MgH_2_ (Lin et al., 2015), which can lead to reduced dehydrogenation temperature and activation energy because of the formation of new Mg-Ce-F species on the surface of MgH_2_. Gulicovski (Gulicovski et al., 2012) et al. prepared MgH_2_-CeO_2_ composite by ball milling of MgH_2_ and nano-CeO_2_ particles. The dehydrogenation activation energy is reduced to 60 ± 10 kJ/mol, indicating that the activation energy is sufficiently decreased by the catalytic effect of vacant CeO_2_ particles. We also developed a new symbiotic CeH_2.73_/CeO_2_ nano-catalyst, which was *in-situ* produced by controlling hydrogenation and oxidation treatments upon the amorphous Mg-Ce-Ni alloys (Lin et al., 2014), leading to significantly improved dehydrogenation performance of MgH_2_-based composite. Moreover, *in situ* TEM and DFT study show that the remarkable catalysis effect is attributed to the spontaneous hydrogen release effect at the interface between cerium oxide and hydride. Because the composite contains only major MgH_2_ but also minor Mg_2_NiH_4_, the effect of CeH_2.73_/CeO_2_ composite on the dehydrogenation properties of Mg_2_NiH_4_ has not been well-understood.

In order to clarify the effect of cerium hydride/oxide composites on the dehydrogenation properties of Mg_2_NiH_4_, in the present study, a series of CeH_2.73_/CeO_2_ composites with different ratios of cerium oxide and hydride were synthesized from pure cerium hydride via controlled oxidation treatments in the air at room temperature, and then they were doped into Mg_2_NiH_4_ by ball milling. The dehydrogenation properties of cerium hydride and cerium oxide-doped Mg_2_NiH_4_ were studied, and the initial dehydrogenation temperature and activation energy were characterized by TG-DSC and a Kissinger's method. Moreover, the phase transitions during dehydrogenation were analyzed through *in situ* XRD experiments.

## Experimental Details

### Materials

The Mg_2_NiH_4_ used in this experiment was prepared by a method of hydrogenation combustion synthesis method (HCS), which was carried out in Prof. Yunfeng Zhu's group in Nanjing Tech University (Gu et al., 2009; Zhu et al., 2017). CeH_2.73_ (purity >99%) was purchased from Hunan Research Institute of Non-ferrous metals. About 0.1 g CeH_2.73_ powder was located in a plastic bottle of 3 ml, in the Ar glove-box. Then it was transferred into the small side box of glove-box. The door of side box was then open to let air in for different time: 0.5, 1.5, 3, 10, and 60 min. The oxidized CeH_2.73_ became CeH_2.73_/CeO_2_ composites, and 2 mol% of CeH_2.73_/CeO_2_ composites were then doped into Mg_2_NiH_4_ by ball milling at 400 rpm for 4 h.

### Characterizations

The phase structures were analyzed by X-ray diffraction (XRD), and the phase transition during dehydrogenation was analyzed by *in situ* XRD analysis. The XRD analysis was carried out by an X-ray diffractometer apparatus (UItima IV, Rigaku, Japan) with Cu-K α (λ = 0.15405 nm). The tube voltage and tube current were 40 kV and 40 mA, respectively. Thermogravimetry (TG) and Differential Scanning Calorimetery (DSC) were used to study the hydrogen desorption behaviors at different heating rates on a METTLER TGA/DSC 3^+^ synchronous thermal analyzer. The microstructures of the Mg_2_NiH_4_-Ce_2.73_/CeO_2_ composites were observed by backscattered electron imaging using scanning electron microscope (SEM) and transition electronic microscopy (TEM).

## Results and Discussion

### Preparation of the CeH_2.73_/CeO_2_ Catalysts

**Figure 1:**
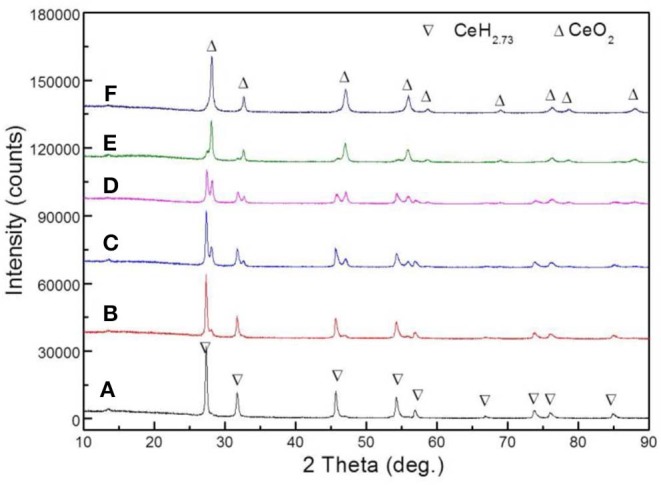
XRD images of six groups of CeH_2.73_/CeO_2_ composite catalysts obtained by oxidation of CeH_2.73_ for different durations: **(A)** 0 min, S1, **(B)** 0.5 min, S2, **(C)** 1.5 min, S3, **(D)** 3 min, S4, **(E)** 10 min, S5, **(F)** 60 min, S6.

**Figure 2:**
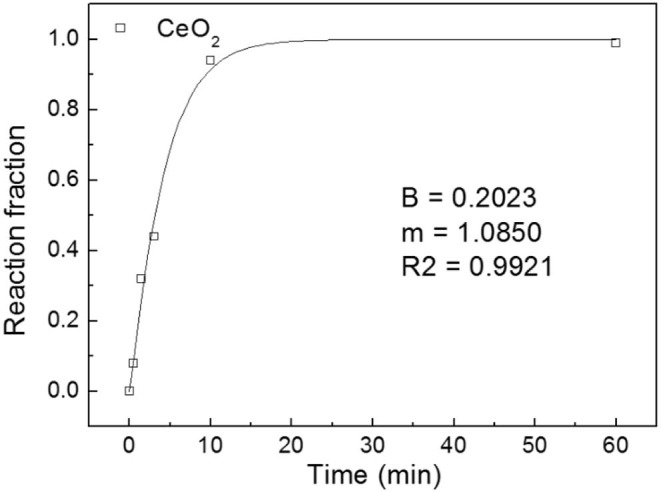
Relative content of the CeO_2_ composites phase as increase of oxidation time.

Six sets of CeH_2.73_/CeO_2_ composites prepared by oxidation treatments for different durations were analyzed by XRD. The CeH_2.73_/CeO_2_ composites are marked as S1, S2, S3, S4, S5, and S6, according to the oxidation duration of CeH_2.73_ of 0, 0.5, 1.5, 3, 10, and 60 min, respectively. The diffraction patterns are shown in Figure 1. With the increase of oxidation time, CeH_2.73_ gradually transforms into CeO_2_. After oxidation of 60 min, no CeH_2.73_ has left. The obtained XRD patterns were refined by a Jade 6.0 software and the phase contents were calculated by the K value (RIR) method. The relative contents of the CeO_2_ phase as increase of the oxidation time are shown in Figure 2. The oxidation data can be fitted by the Avrami–Erofeev equation deduced from the nucleation and growth process:

(1) $$\begin{array}{l} \alpha =1-exp\left(-Bt^{m}\right), \end{array}$$

where α is the ratio of reacted material to total material, m and B are constants. The fitted m is 1.085, which is very close to 1.07, indicating the oxidation of CeH_2.73_ at room temperature is a three-dimensional interface reaction process (Lin et al., 2012b). For the S4 sample, the relative content of CeH_2.73_ is 54.2 wt%, and the relative content of CeO_2_ phase is 45.8 wt%, indicating almost the same molar ratio of cerium hydride and oxide when the oxidation time is around 3 min. After the oxidation treatments, the samples were doped into Mg_2_NiH_4_ by ball milling.

**Figure 3:**
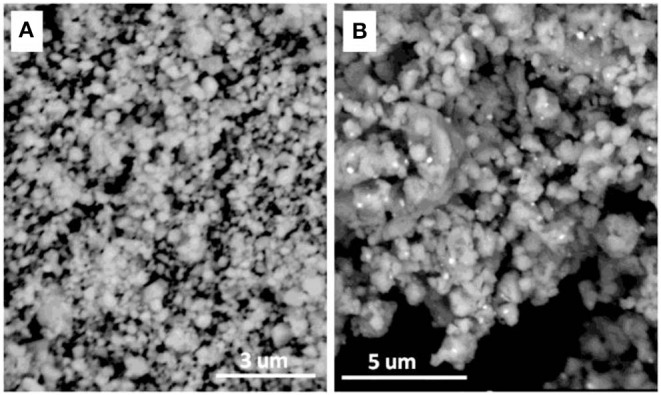
BSEM image of **(A)** the as-oxidized CeH_2.73_/CeO_2_ composite (S5) and **(B)** the ball-milled Mg_2_NiH_4_ + S5 sample.

In order to understand the morphologies of the CeH_2.73_/CeO_2_ composite doped Mg_2_NiH_4_, back scattering scanning electron microscopy (BSEM) was carried out. Because the S1–S4 sample will continue to oxidize in the air, the S5 doped Mg_2_NiH_4_ sample was selected as the experimental material. The morphologies of the as-prepared CeH_2.73_/CeO_2_ composite (S5 sample) and the ball-milled Mg_2_NiH_4_ + S5 sample are shown in Figure 3. It could be clearly seen that the particle size of the as-prepared CeH_2.73_/CeO_2_ composite is about 200–400 nm. After ball milling, the particle size of the CeH_2.73_/CeO_2_ composite is greatly reduced to below 30–100 nm. Figure 3B shows that the CeH_2.73_/CeO_2_ composites, which are brighter particles, are uniformly distributed in the Mg_2_NiH_4_ matrix. The homogeneous CeH_2.73_/CeO_2_ composites could be beneficial for catalyzing hydrogen storage properties for the Mg-based materials (Hong et al., 2009; Shao et al., 2011; Li et al., 2018).

### Dehydrogenation of Mg_2_NiH_4_ + CeH_2.73_/CeO_2_ Composites

**Figure 4:**
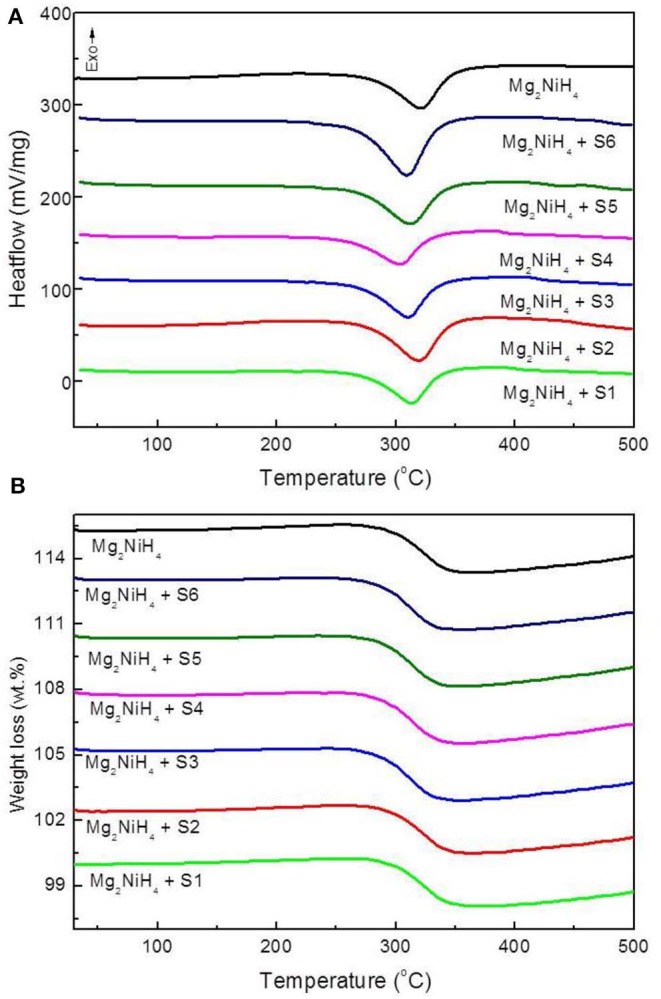
**(A)** DSC and **(B)** TG curves of the ball-milled Mg_2_NiH_4_-CeH_2.73_/CeO_2_ composites materials, at a heating rate of 20 K/min.

The decomposition behaviors of the ball-milled Mg_2_NiH_4_+CeH_2.73_/CeO_2_ composites are studied by TG-DSC synchronous thermal analyzer as shown in Figure 4. The DSC study of Mg_2_NiH_4_ + CeH_2.73_/CeO_2_ composites are at a heating rate of 20 K/min, showing the dehydrogenation initial temperature of Mg_2_NiH_4_ decreases after addition of CeH_2.73_/CeO_2_ composites. Among the six sets of catalysts, the S4 sample obtained by oxidation of CeH_2.73_ for 3 min, exhibits the highest catalytic effect on reducing the dehydrogenation temperature of Mg_2_NiH_4_. The dehydrogenation temperature is decreased to 267°C, which is about 17°C lower than the that of as-milled Mg_2_NiH_4_ (284°C).

**Figure 5:**
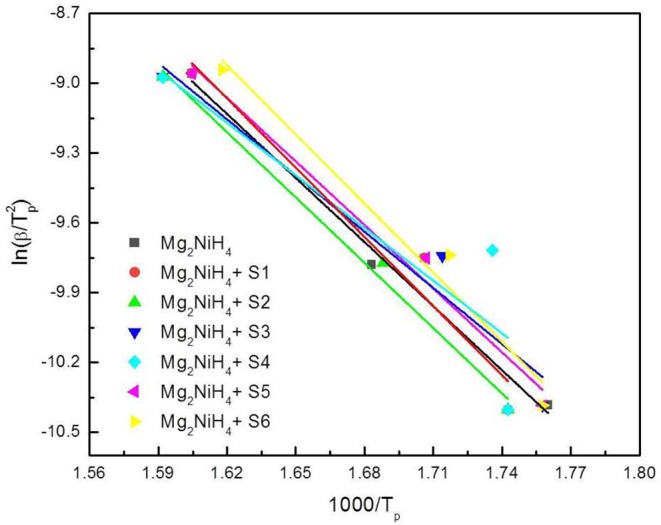
Kissinger's Plot of Mg_2_NiH_4_-CeH_2.73_/CeO_2_ composite system.

To further elucidate the dehydrogenation activation energy of the Mg_2_NiH_4_-CeH_2.73_/CeO_2_ composites, DSC experiments for the Mg_2_NiH_4_ + CeH_2.73_/CeO_2_ composites at heating rates of 10 K/min and 50 K/min were carried out (results not shown). The activation energy of the dehydrogenation process was calculated by using the Kissinger's method (Kissinger, 1957),

(2) $$\begin{array}{l} \text{ln}\left(\frac{\beta }{T_{m}^{2}}\right)=\frac{E_{ds}}{RT_{m}}+C \end{array}$$

where *E*_*des*_ is the dehydrogenation activation energy, *T*_*m*_, β, R, and C are the peak temperature, heating rate of DSC experiments, gas constant and another constant, respectively. The relation between *T*_*m*_ and β is linearly fitted as plotted in Figure 5, and the activation energy was summarized in Table 1. Results show that the S4 sample by oxidation of CeH_2.73_ for 3 min exhibits the best catalytic effect on reducing the dehydrogenation activation energy of Mg_2_NiH_4_ (from 76.3 kJ/mol to 62.6 kJ/mol). Zhang (Zhang et al., 2018) et al. suggested that alloying Mg_2_Ni with Ti, V, Fe, or Si could reduce the hydrogenation and dehydrogenation activation energies to 60–70 kJ/mol. Our study suggests that the CeH_2.73_/CeO_2_ composites are also efficient to reduce the dehydrogenation activation energy of Mg_2_NiH_4_.

**Table 1:** Initial temperature, peak temperature and activation energy of dehydrogenation for the ball-milled Mg_2_NiH_4_ + CeH_2.73_/CeO_2_ composites (20 K/min).

**Materials**	**Initial temperature (^**°**^C)**	**Peak temperature (^**°**^C)**	**Activation energy (kJ/mol)**
Mg_2_NiH_4_	284	320	76.3
Mg_2_NiH_4_ + S1	283	317	82.4
Mg_2_NiH_4_+ S2	283	323	77.8
Mg_2_NiH_4_+ S3	276	315	66.9
Mg_2_NiH_4_+ S4	267	308	62.6
Mg_2_NiH_4_+ S5	276	317	75.7
Mg_2_NiH_4_+ S6	275	314	82.3

Combining XRD and TG-DSC study, it indicates clearly that the initial dehydrogenation temperature and activation energy of Mg_2_NiH_4_ are maximally reduced as the cerium hydride and cerium oxide are in the same amount. These results well-accord with our previous finding on the effect of CeH_2.73_/CeO_2_ composites on the dehydrogenation properties of MgH_2_ (Lin et al., 2014), which means the CeH_2.73_/CeO_2_ composite with the same molar ratio of cerium hydride and oxide could be a good catalyst for the dehydrogenation properties of Mg-based hydrogen storage materials, including both MgH_2_ and Mg_2_NiH_4_.

### Microstructures of the Mg_2_NiH_4_ + CeH_2.73_/CeO_2_ Composite

**Figure 6:**
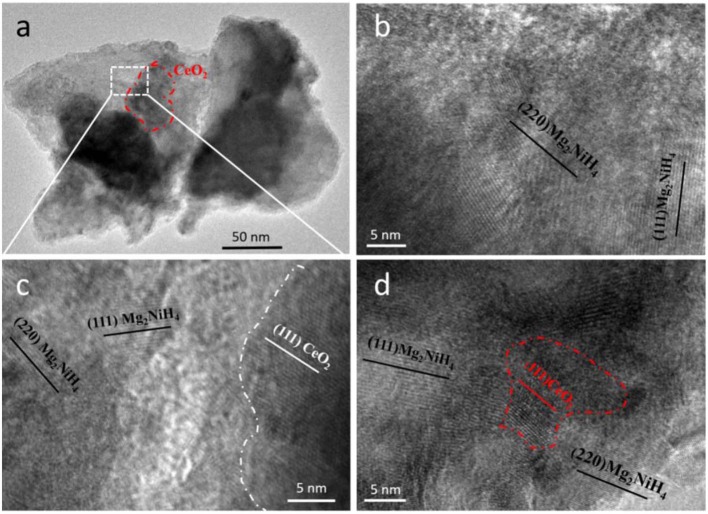
**(a)** TEM and **(b–d)** HRTEM images of the Mg_2_NiH_4_ + S5 composite. **(b)** shows the Mg_2_NiH_4_ matrix, **(c)** shows the magnified interface between Mg_2_NiH_4_ and CeO_2_, **(d)** shows a CeO_2_ particle with size of 5–10 nm.

The morphology and microstructure of Mg_2_NiH_4_ and as-oxidized 10 min CeH_2.73_/CeO_2_ composite (S5) are shown in Figure 6a. From the HRTEM images in Figure 6b, it could be found that the composite matrix is Mg_2_NiH_4_, which lattice fringe corresponds to the lattice plane of (111) and (220). Particle sizes of the CeO_2_ nanoparticles are in a large range from 50–200 to 5–10 nm, which have been clearly shown in Figures 6a,d, respectively. Moreover, it could be seen that the *in-situ* generated CeO_2_ particles are embedded homogeneously in the Mg_2_NiH_4_ matrix after ball milling. The interfaces between Mg_2_NiH_4_ and CeO_2_ composite, as shown in Figures 6c,d, are contacted closely with each other. The homogeneously distributed catalysts can lead to high catalysis effect, and thus be beneficial for the hydrogenation/dehydrogenation properties of Mg_2_NiH_4_. Because of the too small amount of CeH_2.73_, we could not clarify their microstructures in the TEM study.

### *In situ* XRD of Dehydrogenation

**Figure 7:**
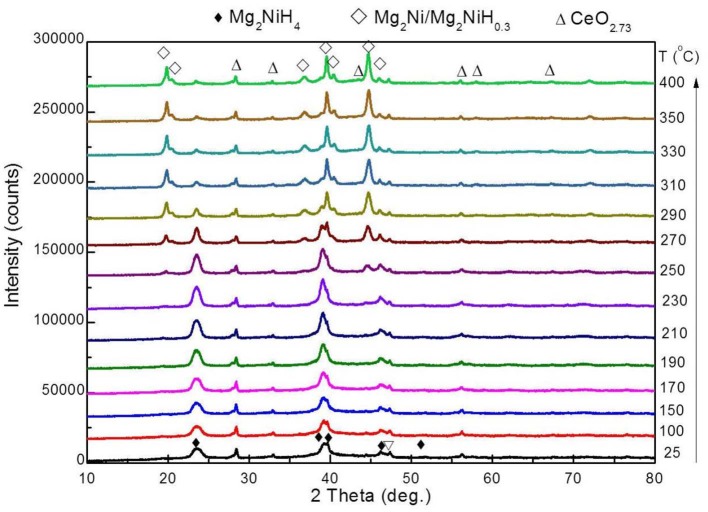
*In situ* XRD patterns of dehydrogenation of the ball-milled Mg_2_NiH_4_ + S5.

The phase transition of the Mg_2_NiH_4_ + S5 sample during dehydrogenation was further studied by *in situ* XRD. The obtained *in situ* XRD patterns are shown in Figure 7. The XRD pattern of Mg_2_NiH_4_ + S5 sample at room temperature contains the diffraction peaks of Mg_2_NiH_4_ and CeO_2_ because the XRD intensity of CeH_2.73_ is too weak. The diffraction peaks of CeO_2_ do not obviously change during the whole dehydrogenation process, indicating that the CeO_2_ phase might act as a catalyst of Mg_2_NiH_4_ dehydrogenation. When the temperature was raised to 250°C, the diffraction peak of Mg_2_NiH_0.3_/Mg_2_Ni phase appears, which means the dehydrogenation of Mg_2_NiH_4_ starts. As temperature increases, the intensity of Mg_2_NiH_0.3_/Mg_2_Ni phase gradually enhances, and that of the Mg_2_NiH_4_ peaks decreases. As temperature reaches about 330°C, the diffraction peaks of CeO_2_ remains unchanged and no MgO is found.

The *in situ* XRD patterns are then refined using Jade 6.0, and the relative contents of phases are calculated by the RIR method. At 270°C, the relative content of Mg_2_NiH_0.3_ phase is 47.1 wt%, and the relative content of Mg_2_NiH_4_ phase is 52.9 wt%. After heating to 290°C, the relative content of the two phases changes rapidly, and the relative content of Mg_2_NiH_0.3_ phase increases to 81.8 wt% and Mg_2_NiH_4_ The relative content of the phase was reduced to 18.2 wt%. Subsequently, the rate of phase change during the heating process was significantly slowed down. The relative content of Mg_2_NiH_0.3_ phase was 89.1 wt% at 400°C, and the relative content of Mg_2_NiH_4_ phase reduces to about 10.9 wt%.

## Conclusion

In summary, CeH_2.73_/CeO_2_ composites with different proportions of cerium hydride and oxide are synthesized from pure cerium hydride via oxidation treatments in the air at room temperature. Oxidation time of 3 min leads to formation of CeH_2.73_/CeO_2_ composite with the same molar ratio of cerium hydride and oxide, which maximally reduces the initial dehydrogenation temperature and activation energy of Mg_2_NiH_4_. The CeH_2.73_/CeO_2_ composite with the same molar ratio is a good catalyst for reducing dehydrogenation temperatures of Mg-based materials.

## Data Availability Statement

The raw data supporting the conclusions of this article will be made available by the authors, without undue reservation, to any qualified researcher.

## Author Contributions

KW and DC: methodology and writing - original draft. KS and TX: methodology and formal analysis. PZ: resources and supervision. WL: supervision and project administration. H-JL: conceptualization, resources, writing - review and editing, supervision, and funding acquisition.

### Conflict of Interest

The authors declare that the research was conducted in the absence of any commercial or financial relationships that could be construed as a potential conflict of interest.
